# Contamination in Observational Research on Child Maltreatment: A Conceptual and Empirical Review With Implications for Future Research

**DOI:** 10.1177/10775595231224472

**Published:** 2023-12-26

**Authors:** Chad E. Shenk, Kenneth A. Shores, Nilam Ram, John M. Felt, Ulziimaa Chimed-Ochir, Anneke E. Olson, Zachary F. Fisher

**Affiliations:** 1 Department of Human Development and Family Studies, 8082 The Pennsylvania State University, University Park, PA, USA; 2 Department of Pediatrics, 8082 The Pennsylvania State University College of Medicine, Hershey, PA, USA; 3 5972 School of Education, The University of Delaware, Newark, DE, USA; 4 Department of Communications, 6429 Stanford University, Stanford, CA, USA; 5 Department of Psychology, 6429 Stanford University, Stanford, CA, USA; 6 The Center for Healthy Aging, 8082 The Pennsylvania State University, University Park, PA, USA

**Keywords:** contamination, child maltreatment, observational research, prospective, retrospective

## Abstract

Contamination is a methodological phenomenon occurring in child maltreatment research when individuals in an established comparison condition have, in reality, been exposed to maltreatment during childhood. The current paper: (1) provides a conceptual and methodological introduction to contamination in child maltreatment research, (2) reviews the empirical literature demonstrating that the presence of contamination biases causal estimates in both prospective and retrospective cohort studies of child maltreatment effects, (3) outlines a dual measurement strategy for how child maltreatment researchers can address contamination, and (4) describes modern statistical methods for generating causal estimates in child maltreatment research after contamination is controlled. Our goal is to introduce the issue of contamination to researchers examining the effects of child maltreatment in an effort to improve the precision and replication of causal estimates that ultimately inform scientific and clinical decision-making as well as public policy.

## A Conceptual and Methodological Introduction to Contamination

A foundational assumption in modern research design and statistical modeling is that units assigned, recruited, or otherwise classified into different levels of a causal variable are and remain mutually exclusive throughout the entire period of study. That is, none of the units assigned to one level of the causal variable (e.g. “control”) participate in or receive a different level of the same variable (e.g. “treatment”). The assumption of creating and maintaining mutually exclusive conditions is implicit in multiple counterfactual frameworks for establishing causal inference in the behavioral sciences (Morgan et al., 2009) but stated outright in the stable unit treatment value assumption (SUTVA) of the potential outcomes framework (Rubin, 2005). Adhering to SUTVA requires that each unit does not receive multiple levels or different versions of the causal variable of interest (see Imbens & Rubin, 2015, p. 10), helping ensure that causal estimates of between-group differences are accurate and unbiased. However, many research studies must contend with SUTVA violations, creating the potential to introduce bias in the direction, significance, or magnitude of between-groups differences because some units have received or been exposed to multiple levels of the causal variable of interest.

One common SUTVA violation is contamination, which is “the use of the treatment by individuals in a control arm” (Cuzick et al., 1997, p. 1017) or “when intervention-like activities find their way into the control group” (Delgado-Rodríguez & Llorca, 2004, p. 640). In experimental research, such as a randomized controlled trial (RCT), contamination occurs when units allocated to a control arm ultimately end up exposed to the treatment under investigation. For example, an RCT examining the efficacy of prostate-specific antigen screening in the prevention of prostate cancer-related mortality may find that a certain number of individuals assigned to a no-screening control condition ultimately received screening from an independent physician (Roobol et al., 2009). Contamination breaks the advantages of random assignment and results in improperly constructed counterfactuals because units in the control arm that received or were exposed to the treatment are now misclassified but retained as controls. When this type of misclassification occurs, the treatment effect inferred from an intent-to-treat analysis will be attenuated relative to the treatment effect inferred from the subset of control units that did not receive the treatment because observed values of the outcome for the control condition are now closer to the observed values for the treatment condition (Angrist et al., 1996; Hirano et al., 2000). Contamination therefore diminishes the statistical power to detect treatment effects (Jo, 2002) as well as the magnitude of those effects (Kerkhof et al., 2010). For the most part, contamination has been studied and solved in experimental research, where it is possible to capitalize on the randomization process as an instrumental variable for obtaining the local average treatment effect (LATE; Angrist et al., 1996), which estimates treatment effects relative to those control units that did not receive the treatment. However, there are far fewer solutions when contamination occurs outside of experimental research, such as when observational designs are needed to estimate between-group differences.

The threat of contamination and the lack of available solutions to address it in observational research is particularly relevant for child maltreatment researchers, regardless of whether the research design is cross-sectional, longitudinal, prospective, or retrospective, simply because random assignment to varying levels of child maltreatment status or type is unethical. Contamination in child maltreatment research occurs when individuals classified as being in a non-child maltreatment comparison condition were, in fact, previously exposed to child maltreatment or will be exposed to child maltreatment during the longitudinal course of a study. Current estimates derived from official case records indicate that 15.4%–65.1% of comparison units within existing child maltreatment cohorts were ultimately exposed to child maltreatment (Scott et al., 2010; Smith et al., 2008; Shenk et al., 2022; Trickett et al., 2011; Widom & Morris, 1997). When it occurs, contamination results in misclassification that introduces measurement error in the binary classification of child maltreatment status or type for individuals in the comparison condition. This measurement error truncates the significance and magnitude of between-group differences of child maltreatment effects by including misclassified comparison units in the statistical model, similar to what occurs in an intent-to-treat analysis of an RCT containing contamination.

**Figure 1.**
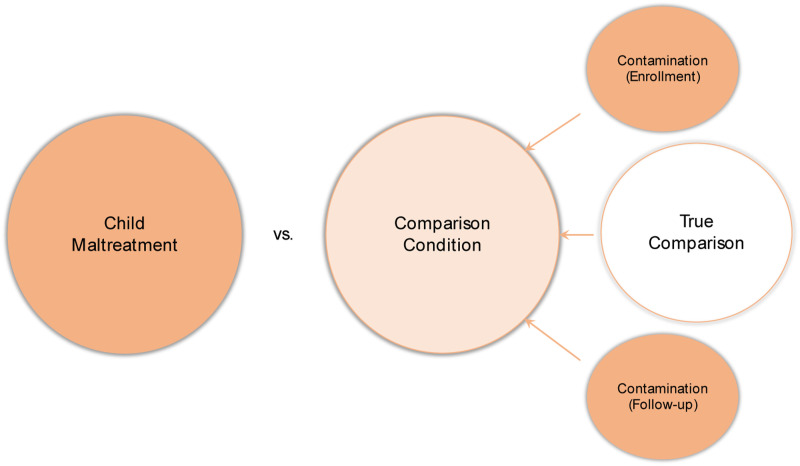
Estimation of Child Maltreatment Effects in the Presence of Contamination. Darker colors represent more severe levels of an observed outcome, lighter colors represent less severe levels. Between-group differences in mean outcome levels are typically modeled where observed values for contamination at enrollment and observed values for contamination during follow-up are combined with observed values from true comparison units (no contamination), elevating the overall mean level of the outcome for the whole comparison condition. Inclusion of misclassified units in the overall comparison condition results in mean values that more closely approximate mean levels of the outcome in the child maltreatment condition, attenuating the significance and magnitude of between-group differences. Instead, child maltreatment researchers are typically interested in modeling the effects of child maltreatment on observed levels of an outcome relative to true comparison (correctly classified) units only.

As illustrated in Figure 1, failure to address contamination attenuates the significance and magnitude of mean differences on an outcome because the level or trend for those in the comparison condition more closely approximates the level or trend in the maltreatment condition due to the inclusion of comparison units who were exposed to maltreatment at the time of classification or during follow-up in models of between-group differences. For example, if a child maltreatment researcher was interested in estimating mean-level differences in depressive symptom severity for those exposed to maltreatment relative to those who were not, contamination would increase the mean severity of depressive symptoms in the comparison condition, thereby minimizing between-group differences. Instead, child maltreatment researchers are most often interested in the evaluation of mean differences for a child maltreatment condition relative to a “true” comparison condition that does not contain any contamination, similar to what occurs in a LATE analysis. Ultimately, observed but uncontrolled contamination, as well as unobserved or unknown contamination, can introduce bias by including misclassified comparison units in statistical modeling of between-group differences.

Widespread attention to contamination in observational research on child maltreatment and methods for addressing it are currently lacking. This manuscript addresses this gap by: (1) orienting researchers on how contamination is introduced into child maltreatment research, (2) reviewing the emerging empirical research reporting the prevalence of contamination and bias in causal estimates generated in both prospective and retrospective cohort studies, (3) describing a dual measurement strategy for addressing contamination, and (4) listing multiple statistical approaches for estimating causal effects in child maltreatment research after contamination is controlled. Advancing awareness to and methods for addressing contamination is a critical step forward for generating more accurate estimates of the adverse effects of child maltreatment that may ultimately drive both stronger causal inference and public policy.

## How Contamination is Introduced into Observational Child Maltreatment Research

### Studies Using Recruitment and Matching Procedures

Prospective cohort studies are regarded the ideal research design for examining the effects of child maltreatment given the proper temporal sequencing of maltreatment exposure and observed outcomes and the ability to repeatedly assess each over time (Widom et al., 2004). In these designs, mutually exclusive child maltreatment and comparison conditions are typically established using a single method of classification at one point in time, namely, at study enrollment. Individuals recruited to a comparison condition who share the same demographic characteristics as individuals in a child maltreatment condition are highly desired for inclusion in the study because they provide an effective counterfactual in the observational design. For example, demographic matching of comparison individuals in an observational design, such as matching an individual in the comparison condition to an individual in the maltreatment condition based on race, income, age, gender, and/or single-parent households, has long been regarded as an effective strategy for controlling extraneous variability due to these observed confounders so that the unique and unbiased effects of child maltreatment can be estimated (Rubin, 1973; Widom, 1988). While recruitment and matching strategies can create balance among important confounders across child maltreatment and comparison conditions at study enrollment, the demographic characteristics most often used to recruit or match comparison individuals are also well-established risk factors for child maltreatment (Institute of Medicine, 2011). Explicit use of these demographic characteristics to create a matched comparison condition, while advantageous to address confounding, can therefore come at a cost, as it introduces the risk that individuals in the comparison condition have already been exposed to child maltreatment or will be exposed during longitudinal follow-up. If so, the presence of children with a maltreatment history in a non-child maltreatment comparison condition is a SUTVA violation generally and contamination specifically.

### Imprecision in Individual Methods Used to Classify Comparison Conditions

Creation and maintenance of counterfactual conditions in experimental and observational research is a challenging task, although precise instruments and continual adherence monitoring can help address these concerns. A single, precise, gold-standard measure of child maltreatment status, type, dimensions, etc. is an ideal solution, however, as of yet, does not exist. Child maltreatment is most often classified using one of three methods: (1) official case reports, such as those resulting from investigations conducted by child protective services (CPS) agencies and documenting one’s history of exposure to child maltreatment, (2) self-report assessments, such as surveys, interviews, and questionnaires assessing one’s prior subjective history of exposure to child maltreatment, and (3) caregiver reports of a child’s maltreatment history, such as those assessing a current or prior caregiver’s history of committing acts of child maltreatment. The reasons for selecting one method over another are many and generally vary according to unique features or constraints of an individual study, such as whether the design is prospective or retrospective, whether child maltreatment is the primary event of interest or a smaller part of a larger epidemiological study, whether the child is too young to accurately report their own maltreatment history (recall bias), etc. The different methods have distinct advantages as well as disadvantages that, when used in isolation, can result in a failure to detect contamination.

For example, official case reports, including those that substantiate an allegation of child maltreatment, are often selected to establish child maltreatment and comparison conditions because they are generated by personnel within government agencies independent of the investigative team and reflect common procedures used throughout a particular region or country. However, official case reports most often require that another person knows about a suspected instance of child maltreatment and that a formal allegation of that maltreatment is made to CPS, where not all allegations are investigated nor do all investigations result in a substantiated designation of child maltreatment. Thus, official case reports are likely lower in sensitivity in that they do not detect all true cases of child maltreatment (e.g. high false negative rate) and err on the side of higher specificity in that when a positive classification of child maltreatment is made, it is likely true (e.g. lower false positive rate). To illustrate, nationally representative surveys in the U.S. indicate that the past-year incidence of child maltreatment is approximately 152 per 1000 children (Finkelhor et al., 2015), an estimate considerably higher than the 8.4–42.9 per 1000 estimate derived from official case reports (U.S. Department of Health and Human Services, 2022). The same discrepancy holds for caregiver-reported maltreatment, as the incidence of child maltreatment estimated through caregiver-report is comparable to the incidence obtained through self-report (Stoltenborgh et al., 2015). This means using only official case reports to create a comparison condition, a method common in prospective cohort studies, would still likely contain contamination by including comparison units who do not have a substantiated designation of child maltreatment but who would self-report or have a caregiver-report child maltreatment if assessed.

On the other hand, self- and caregiver-report methods are often used because of their ease and efficiency in classifying child maltreatment and comparison conditions, particularly in large, nationally-representative, epidemiological studies. Each of these methods have the potential to be more sensitive than official case reports in that they can detect more true instances of child maltreatment (e.g. lower false negative rate) simply by asking individuals whether they or their child were exposed to maltreatment. However, the validity of self- and caregiver-report measures of child maltreatment has been questioned in part because definitions of maltreatment (e.g. poverty vs. neglect; spanking vs. physical abuse), and therefore resulting item content, vary widely across studies (Mathews et al., 2020; Yoon et al., 2021). Moreover, not all individuals with an established child maltreatment history acknowledge this fact during a self- or caregiver-report assessment. For example, over 40% of those who have an official case report of child maltreatment do not disclose this information when assessed via self-report (Baldwin et al., 2019; Widom & Shepard, 1996). This means comparison conditions established and maintained using only self-report methods, such as when adults retrospectively report instances of maltreatment in childhood, have the potential to contain contamination by including individuals who do not self-report child maltreatment but who may have an official case report of child maltreatment. Similarly, caregivers reporting on their own behaviors may be subject to social desirability (Compier-de Block et al., 2017), where they underreport instances of maltreatment to avoid being viewed harshly by researchers or the potential repercussions of a formal CPS investigation.

Overall, commonly used methods for establishing child maltreatment conditions each have notable limitations when used in isolation, namely by emphasizing sensitivity over specificity and vice versa, that can result in a failure to detect contamination or correctly classify individuals in a comparison condition. Each case constitutes a SUTVA violation in that comparison individuals receive different versions (self-report, caregiver-report, official case report) of the causal variable (child maltreatment).

### A One-Time Assessment of a Longitudinal Phenomenon

Many child maltreatment researchers are interested in whether maltreatment occurs during specified age ranges to test hypotheses about critical or sensitive periods. However, exposure to child maltreatment is a time-varying phenomenon that can introduce contamination over time if it is not detected with repeated assessments. For example, the time scale for when child maltreatment occurs is well-documented, occurring most frequently between birth and age four but continuing to affect many children up to age eighteen (U.S. Department of Health and Human Services, 2022), highlighting the benefit of repeatedly assessing maltreatment, say annually or bi-annually, but perhaps even more frequently earlier in life. This age-related trend also highlights the need to continually assess the presence of child maltreatment in a comparison condition even if there was no observed maltreatment in that same comparison condition at study entry. This phenomenon is particularly relevant in the case of a prospective cohort study using extensive longitudinal follow-up. For example, say a research team is interested in examining the effect of child maltreatment prior to age four on rates of substance use at the transition to adulthood, when rates of substance use and abuse are at their highest age-related levels (Johnston et al., 2020). Even if the prevalence of contamination is zero at study enrollment, this team would need to continually screen for child maltreatment occurring in the comparison condition between ages four to eighteen to detect contamination, as members of the comparison condition will be at continued risk for child maltreatment during longitudinal follow-up (Olson et al., 2021). It would also be important to screen for re-exposure to child maltreatment after age four for those in the child maltreatment condition, as this too could bias estimates of the unique estimation of child maltreatment effects from birth to age four. Thus, establishing child maltreatment and comparison conditions cross-sectionally at study enrollment is insufficient for tracking exposure to child maltreatment over time, thereby increasing the risk of undetected contamination that is then included in statistical models estimating between-group differences.

### An Empirical Review of Contamination Bias in Estimates of Child Maltreatment Effects

To our knowledge, the scope of child maltreatment research demonstrating the bias resulting from contamination involves three well-characterized studies that examined child maltreatment effects on broad domains of adverse health and development. The first is a retrospective cohort study (*N* = 2144) that examined the risk for psychiatric disorders in young adulthood for those who were the subject of an official child maltreatment report (Scott et al., 2010), defined as a CPS agency receiving and investigating an allegation of child maltreatment. Despite creating a comparison condition where no one was the subject of a child maltreatment report, 15.4% of these participants self-reported experiencing maltreatment at some time during childhood. When this contamination was controlled by removing these individuals from statistical modeling, effect size magnitudes (odds ratios) for child maltreatment increased by 22% for any past-year and 32% for any lifetime history of a psychiatric disorder, indicating the degree of bias introduced by contamination. Of note, the risk for several individual psychiatric disorders reached statistical significance only after contamination was detected and controlled in this study.

The second is a multi-wave, prospective cohort study (*N* = 514) that examined change in the risk for global indicators of female adolescent health following exposure to child maltreatment (Shenk et al., 2016), where alleged and investigated child maltreatment was substantiated by CPS. Over 44% of the comparison condition in this study either self-reported or had their own history of substantiated child maltreatment. When this contamination was controlled by removing these individuals from statistical modeling, effect size estimates (relative risks) for child maltreatment increased by 24%–130% across all observed outcomes: obesity, teenage births, past-month cigarette use, and clinical levels of major depressive disorder symptoms. Moreover, only when contamination was controlled did the risk for all four outcomes reach statistical significance. This latter point is important because it demonstrates how controlling contamination can promote replication of reported child maltreatment effects, as prior research has found increased risk for these outcomes (Danese & Tan, 2014; Noll & Shenk, 2013; Widom et al., 2007).

The third study used existing data from a multi-site, multi-wave prospective cohort study (*N* = 1354) of children at risk for maltreatment by examining trajectories of child behavior problems from childhood through adolescence (Shenk et al., 2022). First, effect size differences (*d*) were estimated comparing a confirmed child maltreatment condition, where trained research teams using an established coding system reviewed official case reports and determined that maltreatment occurred, to an unconfirmed child maltreatment comparison condition. To estimate the degree of contamination bias, this study then used longitudinal self-reports of child maltreatment to further classify the unconfirmed child maltreatment comparison condition into misclassified and correctly-classified subgroups. Over 65% of the original unconfirmed child maltreatment comparison condition self-reported exposure to child maltreatment at some point prior to age sixteen. When contamination was addressed via modeling the misclassified subgroup as its own condition, effect size estimates for internalizing and externalizing behavior problems increased by 27.5%–52.6% for those exposed to confirmed child maltreatment relative to the correctly-classified subgroup. This increase in effect size magnitude occurred despite the risks for greater internalizing and externalizing behaviors remaining statistically significant regardless of whether contamination was controlled or not.

These three studies suggest that failure to address contamination biased effect size estimates toward the null across a range of pediatric and adulthood health outcomes, making it harder to reliably detect an effect for child maltreatment by attenuating the significance and magnitude of effect size estimates. The importance of not controlling contamination bias is that it may contribute to replication failures, particularly when contamination prevalence is moderate to high and uncontrolled. Conclusions about the unique effects of child maltreatment can therefore vary within and across studies depending on the degree of contamination present and whether this contamination is detected and controlled in statistical models.

## A Dual Measurement Strategy for Addressing Contamination

Because no one measure of child maltreatment correctly classifies all members of a comparison condition, a dual measurement approach (Brenner & Blettner, 1993) has the potential to add precision back into the establishment and maintenance of mutually exclusive counterfactual conditions by correcting measurement error resulting from contamination. To give an example of how child maltreatment researchers can address contamination in a study, each of the studies reviewed above, including both retrospective and prospective cohort designs, created (study enrollment) and maintained (longitudinal) maltreatment and comparison conditions using official case reports, which emphasize specificity. Then, each of those studies used a second method, retrospective self-reports, to identify child maltreatment in the comparison condition that was not observed using official case reports. Using such a dual measurement approach, where, a comparison condition established and maintained using one method is screened a second time using another method to identify additional units exposed to maltreatment, has the potential to enhance detection of contamination within any one study (Swahn et al., 2006). Indeed, a multi-method approach combining self-report and official case reports is more sensitive to detecting maltreatment in a comparison condition, thereby reducing measurement error and increasing the possibility of obtaining more accurate estimates of between-group differences (Shaffer et al., 2008; Widom, 1988). The same benefit holds when child maltreatment and comparison conditions are first classified using retrospective self-reports of maltreatment but where the comparison condition is screened a second time using official case reports to detect contamination.

Once contamination is detected, finding optimal ways to control it becomes important so that it does not bias resulting between-group differences. While this research continues, child maltreatment researchers have two potential options available to them based on the dual measurement strategy noted above: (1) completely remove those individuals in a comparison condition who were identified as having a child maltreatment history from the statistical model, resulting in an overall decrease in sample size (Scott et al., 2010; Shenk et al., 2016), or (2) create a contamination subgroup and modeling this subgroup as a third, distinct condition or level of the causal variable in statistical models (Shenk et al., 2022). While completely removing units from the model may be appropriate when the prevalence of contamination is low, it may be a disadvantage when the contamination prevalence is high. Conversely, modeling the unique effects of those who were originally classified as comparison units but who were later identified as having been exposed to maltreatment as a distinct, third condition may be useful when the contamination prevalence is high but not when it is low. Until a single, gold standard measure for establishing child maltreatment and comparison conditions exists, a dual measurement approach adopted at the initial point of classification and administered during all waves of data collection during longitudinal follow-up may be a relatively simple research design solution for addressing contamination that can inform different statistical modeling strategies. For now, this appears to have the best potential for restoring SUTVA so that more accurate and unbiased estimates of the causal effect of child maltreatment can be achieved.

Unfortunately, research on the optimal ways to control contamination in child maltreatment research, especially ways that promote internal validity and generalizability, is an emerging area of scientific inquiry. However, one study has demonstrated that a dual measurement strategy for controlling contamination by removing units from the comparison condition and therefore the statistical model actually resulted in a revised comparison condition that more closely reflected U.S. population prevalence estimates for all outcomes assessed, aiding generalizability of child maltreatment effects (Shenk et al., 2016). One approach to addressing contamination that is not recommended is moving misclassified comparison units that were identified using one method (e.g. self-report) to a child maltreatment condition that was classified through a second method (e.g. official case reports). Poor agreement exists between retrospective and prospective reports of child maltreatment (Baldwin et al., 2019) and each of these methods have distinct effects on a variety of health outcomes (Coleman & Baldwin, 2023; Francis et al., 2023), demonstrating they are not interchangeable. The differences across individual methods of determining child maltreatment is likely to enhance detection of contamination given their respective strengths and limitations in addressing measurement error (see above) but also introduce heterogeneity in the estimation of child maltreatment effects given their differential associations with outcomes.

As research on the optimal ways to address contamination matures, a dual measurement strategy that allows for the removal of comparison units or creation of a third level of the treatment variable is one current way for child maltreatment researchers to address contamination that has shown improvements in the significance and magnitude of causal estimates. In other words, for now, it is likely better to address contamination with a dual measurement strategy than not address it at all. With a strategy in hand, child maltreatment researchers are able to employ modern statistical methods that further enhance the causal estimation of child maltreatment effects across a variety of research conditions and hypotheses.

## Two Models for Generating Causal Estimates after Contamination is Controlled

Random assignment to treatment conditions affords several advantages for promoting causal inference, such as creating balance across treatment and control groups in potential outcomes, thereby remedying potential threats due to selection bias and other sources of endogeneity (Imbens & Rubin, 2015; Shadish et al., 2002, p. 248). When randomization is infeasible or unethical, there are new challenges as well as opportunities to apply statistical models that can mimic randomization under the right conditions and with necessary assumptions. While various statistical modeling strategies exist for this purpose (e.g. random intercept cross-lagged panel models), we describe two approaches available to child maltreatment researchers that aim to construct counterfactual conditions for promoting causal estimates in observational child maltreatment research before and after contamination is controlled - highlighting not only the bias attributable to contamination but also generating more accurate causal estimates of between-group differences. We delineate the pros and cons of each approach and specify the proper research conditions for using each to maximize their benefit and application for promoting the accuracy of between-group differences in observational child maltreatment research. Importantly, neither statistical method is likely to generate unbiased estimates of child maltreatment effects without first detecting and controlling contamination.

### Cross-Sectional Designs with Covariates: Propensity Score Methods

Suppose a researcher has a dataset consisting of detailed measurement of post-traumatic stress disorder (PTSD) symptoms, binary indicators of child maltreatment status or type, and a list of covariates. In this context, the simple correlation between child maltreatment and PTSD symptoms is likely confounded by a third variable influencing both exposure to maltreatment and PTSD symptoms. For example, children who have been maltreated may also experience economic hardships and if economic hardships are associated with PTSD symptoms but not included in a statistical model, then the model and corresponding parameter estimates will suffer from omitted variable bias (Fritz et al., 2016). Unlike colliders where a third variable is influenced by both treatment and outcome variables, all confounding variables should be included in models. However, adjusting for all possible confounding variables in a multivariate regression framework is untenable in most observational research on child maltreatment given limited sample sizes.

Propensity score methods are appropriate in this case if there are measured covariates, including confounders, available that make it so that maltreatment is conditionally independent of potential outcomes. Propensity score methods are an optimal choice that can result in covariate balance when randomization is unethical, as in the case of child maltreatment. A propensity score refers to “the conditional probability of assignment to a particular treatment given a vector of observed covariates” (Rosenbaum & Rubin, 1983, p. 41). As an example, propensity scores in child maltreatment research refer to the conditional probability of experiencing maltreatment given a set of covariates (e.g., race, income) and obtained using logistic regression, where maltreatment status is the outcome and the covariates are predictors. Different methods, such as inverse probability of treatment weighting, matching, or entropy balancing, can then be applied to achieve balance on a set of covariates across maltreatment and comparison groups. These approaches allow for the assumption that maltreatment status is as good as randomly assigned, conditional on the variables available. Many recent advancements have been made with these methods, notably, “doubly robust” estimators, which are unbiased if the covariates that are available provide conditional independence of either treatment or outcome (Funk et al., 2011). There is also a growing body of research using propensity score methods in observational child maltreatment research (e.g. Kugler et al., 2019).

The National Survey of Child and Adolescent Well-being-II (NSCAW-II; Dolan et al., 2011) provides an example of how the propensity score method can be useful for generating causal estimates before and after controlling contamination. NSCAW-II is a U.S. national probability sample of maltreatment (*N* = 5872) that sampled children between birth and 17.5 years of age involved in child welfare. NSCAW-II measured a wealth of demographic covariates at study entry that can be used to inform a propensity score modeling approach of the effects of substantiated child maltreatment on a variety of outcomes. For example, propensity score matching or weighting could be used to achieve balance on a range of covariates prior to estimating the effects of substantiated maltreatment on a specific outcome, say PTSD symptoms. Results from this propensity score model would provide a baseline estimate for substantiated maltreatment without controlling contamination.

NSCAW-II also assessed caregiver-reported child maltreatment using the Parent-Child Conflict Tactics Scale (CTS-PC; Straus et al., 1998) given the young ages for a large proportion of the child participants. This same research team could then screen for contamination by using the CTS-PC as an indicator of caregiver-reported maltreatment in the unsubstantiated child maltreatment comparison condition. Propensity score methods could then again be used to evaluate the impact of substantiated child maltreatment on an outcome after controlling contamination, where comparison units with caregiver-reported child maltreatment are modeled as a third, distinct group. Causal estimates generated from this final model could be compared to those from the baseline model that did not control contamination to evaluate the amount of bias in the significance and magnitude of causal estimates for substantiated child maltreatment after contamination has been controlled.

Of note, covariate quality is very important for propensity score models. Specifically, covariates that occur before exposure to maltreatment are ideally suited for propensity score models as they are least likely to have been affected by maltreatment itself. However, because maltreatment often occurs when children are very young, many covariates available to researchers do not precede maltreatment and would be problematic colliders or “bad controls” (Cinelli et al., 2022). That is, these variables would act as covariates but because they are affected by maltreatment itself, including them as covariates partially controls for the effect of maltreatment, thereby biasing results. In sum, reducing contamination in the data when using matching-type estimators is important. Children who have been maltreated but are not categorized as maltreated in the data may be more likely to be identified as plausible counterfactuals, as these children may have similar socioeconomic conditions (and other covariate features) as children who have been maltreated. Thus, because of contamination, the propensity score approach may worsen the effect of contamination by identifying other non-identified children with a maltreatment history as useful counterfactuals (see Studies Using Recruitment and Matching Procedures above). To our knowledge, this point has not been made in the child maltreatment literature but it is one to acknowledge when determining the potential effect of contamination on causal estimates, even within the propensity score approach.

### Longitudinal Designs with Repeated Sampling of Outcomes: Synthetic Control Methods

Synthetic control methods are a fusion of event-analysis and matching-type estimators. They are like event-analysis methods in that they require time-varying data and repeated measurement of individuals or groups. They are like propensity score methods in that pre-treatment covariates are used to construct a counterfactual (Abadie et al., 2010; Ben-Michael et al., 2018). The critical advantage synthetic control methods yield relative to matching-type estimators is that *pre-maltreatment outcomes* are used, almost exclusively, as covariates. The intuition behind this approach is that the best predictors for an individual’s future outcomes in the absence of treatment are that individuals’ prior outcomes. In observational research on child maltreatment, a synthetic control modeling framework would use repeated measurements of the outcome of interest, prior to maltreatment occurring, as covariates and then identify children with similar levels and trajectories of that outcome from a donor pool of children who never were maltreated. Those comparison children whose outcomes most closely resemble the outcomes of the to-be maltreated child will then be used as counterfactuals.

To illustrate the approach, consider the Longitudinal Studies of Child Abuse and Neglect (LONGSCAN; Runyan et al., 1998), a prospective cohort study of child maltreatment with repeated measurements from birth through age eighteen. LONGSCAN contains repeated measurements of child behavior problems as well as information describing the onset of maltreatment via official case reports. To apply synthetic controls methods here, it is necessary to have measurements of behavioral outcomes prior to and after the onset of maltreatment as well as measurement of behavioral outcomes for comparison children who are never maltreated. The synthetic controls estimator applies an algorithm to construct a set of non-maltreated children that most resemble a maltreated child’s pre-maltreatment behavioral outcomes (level, trajectory). It then assigns a time-invariant weight to these non-maltreated children, giving more weight to those children whose behavioral outcomes most resemble the maltreated child’s. To identify the effects of maltreatment, the estimator simply takes the post-maltreatment outcome of the child who has been maltreated and subtracts the *weighted* average of the post-maltreatment outcome of the non-maltreated population using the algorithmically determined weights. This means, for example, that the LONGSCAN database, with its repeated measurements throughout childhood, could serve as an excellent resource to estimate the effects of child maltreatment occurring at the transition to adolescence on trajectories of behavior problems during adolescence within the synthetic control approach. Variation in child maltreatment effects on these outcomes estimated in the synthetic control method can also be examined before and after controlling contamination, as determined by self-reports in the LONGSCAN comparison condition. Like with propensity score methods, this approach would not only generate the degree of bias attributable to contamination but also produce more accurate causal estimates of child maltreatment effects on behavior problems. It could also provide a relative comparison of contamination bias when it is determined via prospective versus retrospective self-reports of maltreatment within the same sample.

Though synthetic control methods have the desirable property of leveraging longitudinal data (repeated measurement) to build counterfactual conditions that have more in common with the child maltreatment population, these approaches have practical limitations. First, the approach requires that multiple waves of pre-maltreatment data are available. Given that most maltreatment occurs very early in the life of the child, there may be little pre-maltreatment data for a large portion of the maltreated population. The lack of data limits the applicability of these approaches, unless the researcher is able and planful in securing repeated observations of an outcome prior to maltreatment early in life. Second, even in cases where maltreatment occurs later in childhood, synthetic control methods require multiple repeated measurements after maltreatment. Most studies of child maltreatment have not obtained those data but those that have or do are well-suited for this modeling approach. Third, as with propensity score methods, synthetic control methods require overlap in the covariate distribution, which, in the case of synthetic controls, means overlap of pre-maltreatment outcomes. When children who have been maltreated have very different levels and trajectories of pre-maltreatment outcomes, then synthetic control approaches will struggle to find appropriate comparison units. This concern has been obviated somewhat by recent augmented synthetic controls methods, which use de-biasing approaches to compensate for poor match quality (Ben-Michael et al., 2021). In future studies, as more and more repeated measurements of outcomes of interest are collected prior to an occurrence of child maltreatment, the synthetic control methods will gain value.

### Data Simulations to Extensively Model Contamination Across Research Conditions

Given larger goals of estimating the degree of bias attributable to contamination across studies and the methods for controlling contamination that provide the most accurate causal estimates under different empirical settings, an analysis of simulated data that both mimics the structures of existing empirical data, such as NSCAW-II and LONGSCAN, as well as extends in directions that might be plausibly invoked in future research efforts, including variations in sample size, contamination prevalence, and missing data, can be conducted. Specifically, longitudinal simulation data sets can be generated that assume the data generating process is known ex ante but that vary in sample size, effect size, relative group proportions, age of maltreatment exposure, and extent of missing data. Results can then be quantified with respect to model convergence, extent of bias, direction of bias, and computation time with response surface analysis of the multi-dimensional space, defined by the differences in sample size, effect size, etc., and used to identify the specific research conditions and methods that afford the best control of contamination. Power curves can be generated for all methods across sample sizes and effect sizes of group differences in level and trend to inform future modeling decisions, such as the number of pre-treatment observations of outcome data needed to identify counterfactuals that provide sufficient power for detecting exposure group differences in synthetic control methods. Brought together, analyses of the empirical and simulated data can provide a robust picture of how to control for contamination in child maltreatment research and what method to use when, directly enhancing researchers’ knowledge of the effects of child maltreatment and the design of future studies.

## Distinctions with Other Concepts or Trends in Child Maltreatment Research

### Other SUTVA Violations in Randomized Controlled Trials Research

Contamination in child maltreatment research is unique when compared to other SUTVA violations common in RCT research, such as spill-over and non-compliance. For example, spill-over occurs when units assigned to a treatment condition disseminate information about the treatment to units in a control condition that in turn affects the level of the outcome of interest (Vanderweele et al., 2013). Contamination is different than spill-over in the case of observational research on child maltreatment in that a child recruited to a maltreatment condition who disseminates information about maltreatment to a child in the comparison condition does not expose that child to actual maltreatment, nor is this dissemination likely to affect the level of the outcome for the child in the comparison condition. Non-compliance occurs when units are randomly assigned to a treatment or control arm but ultimately do not adhere to protocol for that condition, including situations where control units seek out or receive the treatment under investigation (Angrist et al., 1996). While contamination can at times be a special case of non-compliance (Cuzick et al., 1997), it is not appropriate to characterize a child in a comparison condition being exposed to maltreatment as an act of non-compliance particularly when there is no obvious motivation to seek out being maltreated. Contamination in child maltreatment research can be a naturally occurring phenomenon independent of the motivations or behaviors of children in a comparison condition.

### Research Examining Differential Effects of Prospective versus Retrospective Methods

There is conceptual overlap between research examining contamination and independent research examining the effects of prospectively-measured maltreatment after adjusting estimates for retrospective self-reports of maltreatment (e.g. Francis et al., 2023). Within this example, the conceptual similarity exists in attempts to recover the causal effect of prospectively-assessed maltreatment by adjusting, removing, or otherwise controlling the presence of retrospectively self-reported maltreatment. Such independent research is important for understanding the differential risks for multiple health outcomes based on whether maltreatment is characterized prospectively or retrospectively. However, the critical difference is that research on contamination (e.g. Scott et al., 2010) within this example is only concerned with controlling retrospectively self-reported maltreatment in the comparison condition, not in the prospectively-determined maltreatment condition. In other words, determining the prevalence of contamination, the degree of contamination bias, or ways to control it does not require adjusting estimates for all self-reported maltreatment within an entire sample, including self-reported maltreatment in a prospectively-determined maltreatment condition. Prior research (Shenk et al., 2022) has shown that those in a prospectively-established maltreatment condition who also retrospectively self-reported maltreatment had the largest degree of risk for observed health outcomes compared to those who only had a self-report or official case report of maltreatment alone (relative to those without either self-report or official case report). It is possible for individuals in a prospectively determined maltreatment condition to also retrospectively report exposure to child maltreatment and adjusting for that self-reported maltreatment can mitigate an important source of variance in health outcomes.

## Conclusion

Contamination is an inherent part of carrying out observational research on child maltreatment. As it is with RCTs, contamination in child maltreatment research represents a violation of critical assumptions (SUTVA) within the counterfactual model of causal inference (Morgan et al., 2009; Rubin, 2005; Shadish et al., 2002), upon which most modern research designs and statistical methods are based. However, unlike RCT research, attention to and solutions for addressing contamination are underdeveloped in observational research and child maltreatment research specifically. The unique contribution of this paper is to provide the foundation for orienting child maltreatment researchers to the phenomenon of contamination, how it occurs, the prevalence in existing research, the bias it can create in causal estimates, a dual measurement strategy for addressing contamination, as well as advanced statistical methods for generating more accurate estimates of child maltreatment effects once contamination is controlled. The goal is to bring greater attention to this issue that in turn spurs future research optimizing methods for addressing contamination when estimating the effects of child maltreatment.

Continuing to generate estimates of child maltreatment effects without addressing contamination is likely to have significant implications on the causal inferences that can be drawn about the effects of child maltreatment. One, if prior research is correct, failing to control contamination results in variation in the significance and magnitude of causal estimates within and across child maltreatment research that is proportional to the different degrees of contamination in those studies. As noted above, there is a wide range of contamination reported in prior research, which may differentially affect statistical power and effect size magnitudes in the estimation of child maltreatment effects. This is clearly an area for future research that could have implications for the replicability of child maltreatment effects, which is influenced by statistical power (Maxwell et al., 2015) and the ability to reproduce effect magnitudes within a similar confidence interval (Tryon, 2016). Two, varying degrees of contamination, and approaches to controlling it, are likely to influence sample size calculations needed to inform the design of future observational research on child maltreatment. A moderate to high prevalence of contamination, which more likely occurs with samples already at risk for being maltreated, is likely to reduce statistical power, which may require oversampling a particular population of interest. Removing comparison units from statistical models to control contamination may further affect statistical power, though an increase in effect magnitude may offset this. Clearly, further research is needed to more directly inform the design of future research in the context of varying degrees of contamination. Third, failure to address contamination appears to result in attenuated effect sizes, potentially underestimating the “true” magnitude of child maltreatment effects. Such results could influence clinical decision making about the role child maltreatment plays in the onset or course of a particular physical or behavioral health condition as well as the motivation to generate formal policy on preventing the adverse health effects of child maltreatment. Concerted and programmatic research on contamination in child maltreatment research is likely to provide needed guidance on each of these implications.
